# The weight of words: Language and ethics in pediatric palliative care

**DOI:** 10.1017/S1478951525101077

**Published:** 2025-10-27

**Authors:** Haley Sherburne, Brenda Schiltz

**Affiliations:** 1 Pediatric and Adolescent Medicine, Mayo Clinic, Rochester, MN, USA; 2 Department of Pediatric and Adolescent Medicine, Mayo Clinic Rochester, Rochester, MN, USA; 3 Department of Internal Medicine, Division of Palliative Medicine, Mayo Clinic Rochester, Rochester, MN, USA

## Reflection

A young man – who loved fishing, adored football, and dreamed of becoming an engineer – lay dying in the intensive care unit as leukemia and infection devastated his body. Confronted with the unimaginable, his parents chose to implement a Do Not Resuscitate (DNR) order to protect him from bruising and rib fractures at the end of life.

A teenage girl – passionate about nail art, hunting, and photography – was nearing the end of life after a prolonged struggle with metastatic osteosarcoma. Her parents made a different choice: they opted not to implement a DNR policy. They needed to know that everything was done to save their daughter.

### Whose parent made the right decision?

Language carries weight. As palliative care clinicians, we understand that language is never neutral. It shapes the framework of our conversations, subtly influences decision-making, and affects how families interpret medical recommendations. The phrase “protecting children from CPR,” though often well-intentioned, highlights the potential physical burdens of resuscitative efforts in terminally ill pediatric patients. Yet it also risks implying that families who do not choose DNR/DNI orders are somehow failing their child or exposing them to unnecessary suffering. Such language can moralize deeply personal and ethically complex decisions.

This is just one example. Other commonly used phrases can also subtly distort how families interpret their choices. Characterizing palliative care as “the regret prevention service” suggests that, through the right conversation, we can somehow eliminate grief or decisional remorse – a reductive and misleading notion. Similarly, using “goals of care” as a catchall phrase for resuscitation status can blur the line between a patient’s broader values – like spending quality time with loved ones – and technical decisions like intubation or chest compressions. Even a seemingly benign question such as, “Do you want to be here for this?” may fail to acknowledge the nuanced interplay of love, obligation, and anticipatory grief that families often navigate.

In palliative care, the words we choose matter. Despite this, we often rely on vague or euphemistic language to describe prognosis. Words like “grave,” “grim,” “poor,” or “incurable” imply serious illness but fail to convey specific timelines or expectations (Kelemen et al. 2017). While this may stem from a desire not to upset the family or child (Aydın et al. 2023), this imprecision can unintentionally influence decision-making. These words approximate the truth, and they also convey judgment and bias. We should be cautious when using such adjectives to describe a prognosis – even when they come from a place of kindness or clinical uncertainty.

These findings emphasize the complexity of end-of-life communication. How do we move forward? How do we use language that is clear yet compassionate, informative without coercion, and supportive without presumption?

Serious illness conversations have evolved significantly over time (Sisk et al. 2016). Our language must evolve alongside them. Communication training has been shown to improve patient experiences (Fallowfield et al. 2003; van Dulmen and Holl 2000) by enhancing relational connection, information clarity, and conversational competence (Davis and Bass 2025; English et al. 2022) – all qualities that children and families identify as essential in clinical encounters (Hsiao et al. 2007). Relational connection is a key component of the ethics of care framework, which recognizes that serious illness conversations are deeply relational acts, grounded in mutual vulnerability, empathy, and moral attentiveness (Bertaud et al. 2025). Recognizing the interconnectedness of patients, families, and clinicians allows for care that honors both autonomy and dependence, and reframes serious illness dialogue as a shared human experience rather than a purely clinical task.

Language is a tool. It can build trust, convey nuance, and foster meaningful connection. But it can also mislead, stigmatize, and unintentionally burden families with guilt or fear. There is no universal script – no single phrasing that suits every family or situation. Still, by leaning into honest and unembellished communication, we can offer families clarity, dignity, and respect. In doing so, we invite them into shared decision-making and avoid the unintended consequences that language, even when well-meaning, can bring. In palliative care, this is not just good communication – it is ethical practice.

There is no instruction manual for life and death in childhood. In the cases above, both sets of parents made the right decision for their children and their families. As medical professionals, we inevitably bring our own perspectives and biases into these conversations – but our responsibility is to offer objective, nonjudgmental guidance in situations that are often emotionally charged and ethically complex. Ultimately, our role is not to steer families toward a single “right” choice, but to create space for their values, hopes, and fears to be heard. When we speak with clarity and compassion – recognizing the immense weight of our words – we honor not only the medical realities of serious illness but also the deep humanity at its center. This is the heart of palliative care.
